# Comparison of Volar and Dorsal approaches for surgical treatment in fracture of proximal half of the radius

**DOI:** 10.12669/pjms.292.3226

**Published:** 2013-04

**Authors:** Seyed Abdolhossein Mehdi Nasab, Nasser Sarrafan, Mohammd Fakoor, Maghsood Mohammadzadeh

**Affiliations:** 1 Seyed Abdolhossein Mehdi Nasab, Associate Professor of Orthopaedic Surgery, Musculoskeletal and Rehabilitation Research Center, Ahvaz Jundishapur University of Medical Sciences, Ahvaz, Iran.; 2 Nasser Sarrafan, Associate professor Of Orthopaedic surgery, Musculoskeletal and Rehabilitation Research Center, Ahvaz Jundishapur University of Medical Sciences, Ahvaz, Iran.; 3 Mohamma Fakoor , Associate professor Of Orthopaedic surgery, Musculoskeletal and Rehabilitation Research Center, Ahvaz Jundishapur University of Medical Sciences, Ahvaz, Iran.; 4 Maghsood Mohammadzadeh, Orthopaedic Resident, Musculoskeletal and Rehabilitation Research Center, Ahvaz Jundishapur University of Medical Sciences, Ahvaz, Iran.

**Keywords:** Radius fracture, Volar approach, Dorsal approach

## Abstract

***Objective:*** Fracture of the proximal half of the radius shaft can be exposed by either one of volar or dorsal approaches. The aim of this study was to compare the results of volar and dorsal approach for surgical treatment of proximal half fracture of the radius.

***Methodology: ***This prospective study was performed from April 2008 to March 2012 in two teaching hospitals. Seventy adults patients with closed fracture in proximal half of the radius or radius and ulna were operated on and fixed using small plate and screw by volar approach (VA) (39 patients) and dorsal approach (DA) (31 patients). Comparison of the results in both surgical approach were the primary measurement outcome. Duration and time of procedure, rate and time of fracture union and motion of the forearm were assessed at 4 months after operation.

***Results:*** Mean age of the patients with VA and DA was 25.3 and 26.5 years respectively. There was 26 male and 13 female in VD and 22 male and 9 female in DA patients. Radial nerve injury in VA and DA occurred in three and two patients, infection in one and nonunion in one other patient was seen in each group. There was no significant difference in duration of procedure or time of union after both approaches =0.643. Mean rotation of forearm was 135 deg. in VA, and 138 deg. in DA patients at 4 months post surgery.

***Conclusion:*** There was no significant difference in term of fracture union, early complications, and range of forearm rotation between volar and dorsal approach for the fixation of radius fractures in its proximal half.

## INTRODUCTION

Radius and ulna are different from other long bones of the body. These bones are developed for mobility rather than stability. The unequivocal mobility that exists in human upper limbs is due to the unique anatomy of the elbow, forearm, wrist, and hand. Fractures of the adults forearm bone dyaphysis is usually caused by a severe strike, accompanied by displacement and instability due to muscle stretch.^1^^-^^3^

Because of poor results after closed reduction and casting, open reduction and internal fixation (ORIF) using plate and screws is the standard and preferred method for treatment of displaced forearm fractures.^4^^,^^5^ In a study, Anderson concluded that ORIF is the most physiologically compatible type of internal fixation of the forearm fractures.^6^

The surgical approach can be selected according to the type of fracture and soft tissue damage.^7^ For fixation of the ulnar fracture, direct approach on the ulnar side of the bone is usually preferred, while for the fracture of middle and distal third of radius, the anterior approach is employed. For proximal 1/3 or ½, of the radius it is controversial to use either anterior or posterior approaches and each one of them has its own advantages and disadvantages. Anterior approach (Henry) can be used for the whole length of radius shaft, but in cases such as dorsal soft tissue damage which requires debridement, posterior approach is preferred.^1^^-^^3^

This study was conducted for treatment of proximal half of radius fractures at our hospitals to determine if there is a difference between anterior and posterior approaches in terms of complications or fracture union.

## METHODOLOGY

This prospective study was conducted between 2008 and 2011 at two teaching hospitals of Imam Khomeini and Razi in Ahvaz Iran. Inclusion criteria were the following: patients with closed fracture of radius, or radius and ulna (type A or B in AO classification) in proximal half of the forearm, age over 15 years old, no concomitant injuries in the same forearm or hand, and no neurovascular damage. The operation method was explained to the patients and written informed consent was obtained. According to their type of referral, patients were divided into two groups. For the first group with fracture of radius, anterior approach, and for the second group, posterior approach was employed. All patients underwent surgery within 24 hours to 6 days after injury (average 2.3 days). Tourniquet was used. One gram of cephalothin was injected 2 hours before surgery. In the volar approach, incision was made between mobilewad and flexor pronator mass. After protecting the arteries and nerves, fracture was exposed and ORIF with plate and screws (small DCP 3.5mm) was performed.

In the dorsal approach, forearm was in the pronation position and incision was made along the line between lateral Condyle of the elbow toward Lister tubercule. After exposure and protection of posterior interosseous nerve and dividing supinator muscle, fracture was exposed and fixed with the same type of plating. After surgery, a dorsal splint for two weeks was applied and then physiotherapy was recommended for all of the patients. Clinical examination and radiography was performed at 4, 8, and 16 weeks after surgery. The variables considered included: duration of operation, time taken for union, movements of wrist and forearm, and possible complications that were assessed and compared in the last follow-up. The criterion for union was observation of callus formation in 3 cortexes in AP lateral radiographs. Goniometry was used for measurement of forearm movements.

***Analysis of statistical data: ***First, cod-sheet was prepared from the data collected, and entered in the SPSS-software version 17. Then, the mean and minimum and maximum standard deviation for quantitative variables, and absolute and relative frequencies of qualitative variables were determined. For comparisons of qualitative statistics Exact Fisher and Chi-square tests, and for quantitative statistics, t-test was used. P-values ≤0.05 were regarded significant.

## RESULTS

Of 91 patients with proximal fractures of the forearm, 21 were excluded from the study due to lack of follow-up, and the remaining 70 patients were evaluated. Of 39 patients in the volar group, nine cases had isolated radius fractures and 30 cases had both radius and ulna fractures. The dorsal group consisted of 31 patients, of whom 7 had radius and 24 had fractures of both bones. Other details of patients can be seen in Table-I. AO classification was used for classifying fractures.

**Table-I:** Demogrophic data and Resalts in both groups

*Variable*	*Volar APP*	*Dprsal APP*	*PV*
Count	n=39	n=31	p=0.7
Age	mean SD	mean SD	
	25.31± 7.34	26	
*Type Fx*			
A	N=20	N=22	
B	N=11	N=9	P=0.9
Duration of procedure	67.3(45-105)	62.6(50-90)	P=0.03
Union Time (weeks)	15.69 ±3	15.74±2.8	P=0.09
Rata of union	97.4%	96.8%	P=0.09
Infection	n=1(2.6%)	n= 1(3.2%)	P=0.85
Nerve injury (Radial nerve)	n=3(7.7%)	n=2(6.5%)	

**Table–II:** Range of motion of the forearm

*Rom(deg)*	*Volar APP*	*Dorsal APP*	*p value*
*Forearm Rotation*	*n= 39*	*n=3*	
	*Mean (SD)*	*Mean (SD)*	
At 1 month	86.15±15.06	96.29±10.56	P=0.0.2
At 2 month	122.31±20.45	125±18±11.50	P=0.44
At 6 month	135.6±11.13	138.87±7.03	P=0.16

The mean duration of surgical procedure in volar and dorsal approach was 67.3 minutes (45-105 minutes), and 62.6 minutes (50-98 minutes) respectively, that shows no significant difference (P=0.003). Mean time taken for union, degree of union and non-union, and also complications are shown in Table-II. In the dorsal approach group, two cases of radial nerve injury occurred, of whom, full recovery in one patient and partial recovery in the other one was seen after four months. In the patients with volar approach, injury to the sensory branch of radial nerve in one patient weakness in extension of the thumb and fingers was present in two patients at last follow-up.

## DISCUSION

While volar approach is the standard and preferred method for fracture of distal half of radius, but for proximal half is controversial.^8^ Generally, the employed method must be safe, and have the least risk of damage to the neurovascular tissues. Furthermore, surgeon must be more skillful and familiar with it. In the dorsal approach, access to the bone is easier and the posterior or tension surface of the bone is in full view, making it more suitable for the placement of the plate. However, there is the possibility of damage to the PI nerve.^9^ In this study, patients of the two groups did not differ much in terms of age, gender, and type of fractures, and although duration of the operation was longer in the volar group than that in the dorsal group, this difference was not significant p=0.004. To date, there have been only a few studies on comparison of the volar and dorsal approaches for the proximal radius fractures.

Cross et al performed a study on 10 fresh-frozen adult cadaveric upper-limb specimens. They found the two approaches did not result in a significant difference in area exposed and concluded that depending on case requirements, either the dorsal or volar approach will provide adequate exposure to the proximal radius.^10^ Kwansy et al studied 80 patients with proximal radius fractures operated by dorsal approach.^8^ They reported one case of infection and two cases of damage to the sensory branch of radial nerve which were recovered. In their study, range of approach and good coverage on the plate were reported as benefits of the volar approach. In another study, Bartonicek et al used volar approach for exposure of proximal radius, and reported high rates of union without complications.^11^ Batler et al, comparing dorsal and volar approaches and concluded that dorsal approach was more suitable for proximal radius of up to 2.6 cm distal to elbow joint, so that, damage to the nerve is prevented.^7^ In the anterior approach, incision must be limited to 4.7 cm below the elbow to reduce the risk of vascular injury.

Dietz et al compared fixation of forearm fracture in volar and dorsal approaches and found that mal-positioning of plates occurred more in the dorsal approach which leads to the impaired rotational kinematics of the forearm.^12^ Nasab et al. observed two cases out of 28 patients with forearm proximal fractures treated by dorsal approach had deep radial nerve injury, and partial recovery was achieved in one of the cases.^13^ Our study, in terms of rate and duration of union, was similar to previous studies. In both groups, one case of infection and one case of non-union was observed, which indicates that type of surgical approach does not influence rate of union, duration of union, or occurrence of infection.

In our study, three cases of radial nerve damage were observed after volar group. Two cases had injuries to the sensory branch and one case had damage to the PI nerve branch. Recovery of sensory and motor branch of radial nerve was observed in two patients, but sensory deficit remained in one patient. Damage to the PI nerve has been usually reported in the dorsal approach, but its occurrence after volar approach indicates that, careful attention must be paid during supinator muscle dissection.^14^ In the dorsal group, two cases of damage to PI nerve were observed, with recovery in one patient and no recovery and inability in active extension of the fingers in another patient. Thus, intra-operative nerve exploration must be conducted with sufficient accuracy, particularly in fractures of proximal third, as when dividing supinator muscle, it should be dissected layer by layer to prevent nerve damage.

On the other hand, given the changing position of the nerve with forearm movements, care must be taken with the precise position of the forearm pronation. As in this position, PI nerve is parallel to the radius shaft and by longitudinal dissection, risk of damaging it is reduced.^15^ The impaired functions of the patients in our study were evaluated with the forearm rotational movements, which were measured at the end of the months 1, 2, and 4 after the operation. The interesting point was that limitation of forearm rotational movement in the first few weeks differed considerably in both groups, and in the volar group, it was much more severe. This difference gradually reduced and after four months reached an acceptable and similar level in both groups. Reduction in rotational movement of the forearm in the first month was approximately 90 degrees, which later reached average 36 degrees.

***Limitations of the study: ***This study was conducted at university teaching hospitals, and surgeries were performed by senior residents. The follow-up period was short, but of course the aim was to examine short-term complications until healing of fractures. Therefore, for evaluation of precise hand and forearm functions, studies with longer follow-up periods are necessary.

## CONCLUSION

The results and short-term complications of both volar and dorsal approaches for surgical treatment of proximal radius fractures were similar. Because of the possibility of radial nerve injury with both approaches, great care must be taken during surgical dissection of supinator muscle and exposure or protection of radial nerve. Thus, the experience and skills of the surgeon are important when choosing either approach.

## Authors Contribution:

SA Mehdi Nasab, writing and editing the manuscript. M Mohammadzadeh, designed the study, N Sarrafan, M Fakoor collected the data. The authors did review and final approval of manuscript.
